# Re-Evaluating the Appropriateness of the “Don’t Know” Response Option: Guessing Rate as a Source of Systematic Error on Autism Knowledge Assessments

**DOI:** 10.1007/s10803-024-06452-w

**Published:** 2024-08-03

**Authors:** Camilla M. McMahon, Maryellen Brunson McClain, Cassity R. Haverkamp, Bryn Harris

**Affiliations:** 1 Department of Social and Behavioral Sciences, Miami University, 1601 University Blvd., Hamilton, OH 45011 USA; 2 Department of Counseling and Educational Psychology, Indiana University Bloomington, 201 N. Rose Avenue, Bloomington, IN 47405 USA; 3 South Point Counseling Services, 1206 S Jordan Pkwy STE D, South Jordan, UT 84095 USA; 4 School of Education and Human Development, University of Colorado Denver, 1380 Lawrence Street, Denver, CO 80217 USA

**Keywords:** Autism knowledge, Guessing rate, “Don’t know” response option, Validity, Measurement error

## Abstract

Several autism knowledge assessments include “don’t know” as a response option. The inclusion of this response option may lead to systematic error, such that participants’ guessing rate affects the measurement of their autism knowledge. This study examines both predictors of guessing rate for autism knowledge and predictors of autism knowledge, including guessing rate. School-based professionals (*n* = 396) completed the Autism Spectrum Knowledge Scale Professional Version-Revised (ASKSP-R; McClain et al, Journal of Autism and Developmental Disorders 50(3):998–1006, 2020). and the Autism Stigma and Knowledge Questionnaire (ASK-Q; Harrison et al, Journal of Autism and Developmental Disorders 47(10):3281–3295, 2017). Both assessments include “don’t know” as a response option. Guessing rate was the strongest predictor of autism knowledge across both the ASKSP-R and the ASK-Q assessments. For the ASKSP-R, participants who were school psychologists, practicing for more years, had more autism-related clinical experiences, and who personally knew an autistic person had a higher guessing rate. School psychologists and participants who worked with more autistic students scored higher in autism knowledge. For the ASK-Q, participants with greater self-perceived autism knowledge had a higher guessing rate. Participants with a doctorate degree, who personally knew an autistic person, and who worked with more autistic students scored higher in autism knowledge. Guessing rate can be a source of systematic error on autism knowledge assessments. Potential solutions to correct for guessing rate are examined and recommended for future use.

Autism knowledge applies to a variety of populations including autistic individuals, professionals working with autistic individuals, indirect service providers (e.g., law enforcement officers) who interact with numerous populations including autistic individuals, and parents of children with or at increased likelihood for autism, as well as the broader general public. Adequate autism knowledge within each of these groups is important. Professionals’ autism knowledge is linked to the satisfaction of racially and ethnically minoritized caregivers undergoing the autism evaluation process with their child (Stahmer et al., 2019). Law enforcement officials who have received autism-specific training have reported feeling more prepared to respond to a call involving autistic individuals (Gardner et al., 2019). Autism knowledge is also linked to attitudes toward inclusion and positive classroom practices for general education teachers (Segal & Campbell, 2012). It is also important for the broader general public to understand autism, as increased knowledge may result in autism-friendly attitudes in adults (Jones et al., 2021). If parents of children who are at an increased likelihood of autism are knowledgeable about autism, they may be able to recognize early signs and symptoms, which can then result in earlier identification and service provision (Benallie, 2019). More recent research has also focused on ways to improve autism knowledge and awareness and has shown that trainings, brief videos, and increased interactions with autistic individuals improve autism knowledge and decrease stigma (Gillespie-Lynch et al., 2015; Ha et al., 2022; Mavropoulou & Sideridis, 2014).

Autism knowledge measures aim to objectively capture someone’s actual knowledge and understanding of autism, which can be used to determine current knowledge levels and changes in autism knowledge over time. Accurately measuring autism knowledge is paramount to ensure valid and reliable estimates of the construct and consistency across research and applied contexts. Additionally, it is important to use objective questions to assess actual autism knowledge, as a person’s self-perception of their own autism knowledge may not align with their actual autism knowledge (McMahon et al., 2020). Autism knowledge measures may also be used to evaluate autism knowledge as an outcome in applied contexts including improvements due to interventions, experiences, and professional development, as well as program (e.g., pre-professional preparation) evaluation (Harris et al., 2020; Harrison et al., 2017b). If autism knowledge isn’t assessed accurately, it might lead to poor judgements and limited opportunities for further education. For instance, if professionals have an artificially high autism knowledge score on an assessment, they may be less likely to seek out or may even be discouraged from pursuing professional development training related to autism. Professionals who are overconfident in their autism knowledge may be more likely to make consequential mistakes in their interactions with autistic individuals (e.g., a law enforcement officer may not realize that bright lights and loud sirens can make it difficult for an autistic individual to respond in an emergency; a pediatrician may decide not to refer a child with subtle autistic characteristics for further evaluation). Accurate measurement of autism knowledge is critical for identifying which professional trainings are most effective, as well as which professionals are most likely to benefit from additional autism education opportunities.

## Measuring Knowledge and Lack of Knowledge

In the current literature, several assessments have been developed to measure autism knowledge (Harrison et al., 2017b). As noted by Harrison et al. (2017b), these assessments vary in their response format, with assessments often using a Likert scale, true/false responses, or multiple choice with categorical options. Of particular interest to the current study, some assessments use a response format that constrains participants to choose an answer to the question (e.g., true/false) whereas other assessments use a response format that permits participants to indicate when they are unsure of the answer (e.g., true/false/don’t know). The “don’t know” response option is often used to reduce participants’ random guessing, which may artificially inflate participants’ knowledge scores (McClain et al., 2020).

Although the “don’t know” response option may be intended to improve assessment validity, there are significant concerns about the use of this response option in the broader social science literature (e.g., Miller & Orr, 2008; Mondak, 1999, 2001; Mondak & Anderson, 2004; Mondak & Davis, 2001). Three primary concerns about this response option are as follows:

1. *The “Don’t Know” Option Masks Partial Knowledge*: Often, participants have partial knowledge about a question. For instance, in a multiple choice question, a participant might be able to confidently eliminate one or two response options, but remain uncertain about the ultimate correct answer. In this circumstance, the participant may genuinely not know the final answer to the question, such that the “don’t know” response option is appropriate. However, the use of this option masks that the participant has *some* knowledge about the question and may not allow researchers to differentiate between participants with partial knowledge and no knowledge (e.g., Mondak, 1999).
2. *Guessing Rate Affects Knowledge Score*: When participants are not fully certain of their responses to a question, participants have the option of making an educated guess (possibly receiving credit for a correct response) or indicating that they don’t know the answer (definitely not receiving credit for a correct response). As such, participants’ final scores on a knowledge assessment are partially driven by their tendency to guess or refrain from guessing. Participants who are more self-confident or more competitive, for example, may be more likely to guess, such that knowledge scores are affected by personality traits or other irrelevant individual differences (e.g., Mondak, 1999).
3. *Knowledge Relates to Metacognitive Awareness of Knowledge*: Participants who are more knowledgeable in a content area can more accurately judge the accuracy of their responses. Participants who are less knowledgeable in a content area have more difficulty judging the accuracy of their responses and tend to be overconfident in their responses (e.g., Kruger & Dunning, 1999; McMahon et al., 2020). As such, participants who are more knowledgeable in a content area are better able to recognize the limitations of their knowledge, may be more apt to select a “don’t know” option when appropriate, and therefore may be less likely to have their knowledge score inflated through guesses. Participants who are less knowledgeable in a content area are often overconfident in their knowledge, may be more apt to think they know an answer and guess accordingly, and therefore may be more likely to have their knowledge score inflated through guesses. As such, in a special circumstance of the guessing rate confound discussed previously, participants’ content knowledge may affect their tendency to guess, thereby skewing the measurement of that content knowledge.

Although the “don’t know” response option is usually well-intentioned and designed to provide respondents with accurate response options, it may ultimately decrease assessment validity. When this option is available, a systematic (nonrandom) source of error is introduced into the measurement of content knowledge: participants’ tendency to guess (e.g., Mondak, 1999).

## The Current Study

Although there are several autism knowledge measures with adequate reliability and validity, no research to date has evaluated the impact of response option type on measure quality, specifically the inclusion of a “don’t know” option. To address this gap in the research, the current study examines the impact of the “don’t know” response option on autism knowledge measures. The current study has the following three main goals:

1. To examine predictors of guessing rate for autism knowledge questions to better understand which participants are more likely to guess on autism knowledge questions, rather than indicate that they don’t know the answer.
2. To examine predictors of autism knowledge, with a special focus on whether guessing rate is predictive of autism knowledge.
3. To examine two potential approaches to correct for guessing rate: (a) including guessing rate as a predictor variable when evaluating autism knowledge and (b) randomly assigning a response to any autism knowledge questions which participants skipped or didn’t know the answer.

In the current study, we assess autism knowledge in a sample of school-based professionals using two psychometrically-supported autism knowledge questionnaires, the Autism Spectrum Knowledge Scale Professional Version-Revised (ASKSP-R; McClain et al., 2020) and the Autism Stigma and Knowledge Questionnaire (ASK-Q; Harrison et al., 2017a). School-based professionals are an ideal sample for this study, as such professionals tend to have a wide range of educational and clinical experiences related to autism. While some school-based professionals have had extensive experience working with autistic students, other school-based professionals have had limited firsthand experience with students with autism.

## Method

### Participants

A total of 567 school-based professionals (audiologists, speech-language pathologists, and school psychologists) participated in this study. Participants were recruited via audiology, speech language pathology, and school psychology state professional organizations. These specific disciplines were chosen because of their involvement in school-based service provision for students receiving special education services and their historical involvement in service provision for autistic individuals broadly. Researchers contacted member(s) of the identified professional organization leadership team (e.g., president, research chair) and asked them to share the study information, including participant inclusion criteria and the link to the Qualtrics survey, with their membership electronically. Interested members read information about the study, which had been declared exempt by the Institutional Review Board, and provided consent electronically prior to accessing survey content. Immediately following consent, participants completed screening questions to ensure they met inclusion criteria; participants who endorsed any items not in alignment with inclusion criteria were removed from the sample.

From the initial sample, 171 participants were excluded for the following reasons: 17 participants did not identify as an audiologist, speech-language pathologist, or school psychologist; 11 participants only had a bachelor’s degree and therefore were not independently practicing school professionals; 47 participants did not primarily practice in a school setting; 1 participant did not reside in the United States; 75 participants did not complete any autism knowledge questions; 19 participants had missing data on one or more predictor variables for the regressions (see data analyses for more information); and 1 participant showed an irregular response pattern on both autism knowledge questionnaires. An autism knowledge questionnaire was deemed to have an irregular response pattern if the participant gave a “don’t know” response or did not provide an answer to 80% or more of the autism knowledge questions. As such, the final sample consisted of 396 participants. Two participants had an irregular response pattern for the ASKSP-R assessment (McClain et al., 2020) only, and these participants were excluded from data analyses related to this questionnaire (*n* = 394). Seven participants had an irregular response pattern for the ASK-Q assessment (Harrison et al., 2017a) only, and these participants were excluded from data analyses related to this questionnaire (*n* = 389).

Approximately half of the study sample identified as school psychologists (*n* = 199), and approximately half of the sample identified as audiologists or speech-language pathologists (*n* = 197). The majority of the sample was female (*n* = 363), had earned a master’s or specialist’s degree as their highest degree (*n* = 334), and personally knew someone with autism (*n* = 324). See Table 1 for more detailed information regarding participant demographics.

**Table 1:** Participant characteristics and descriptive statistics

Variable	*n*	%	M	SD	Range
Discipline					
Audiology or speech-language pathology	197	50			
School psychologist	199	50			
Gender					
Female	363	92			
Male	33	8			
Degree					
Master’s or Specialist’s	334	84			
Doctorate	62	16			
Personally Knowing Someone with Autism					
Personally Know Someone with Autism	324	82			
Do Not Personally Know Someone with Autism	72	18			
Years Practicing			13.28	10.65	0–50
Perceived Autism Knowledge			7.52	1.38	3–10
Confidence Providing Services to Autistic Students			7.35	1.63	1–10
Educational Experiences Related to Autism^			2.10	0.42	1-3.50
Practical/Clinical Experiences Related to Autism^			1.99	0.49	1–4
Number of Autistic Students Worked With^			4.59	1.75	2–7
ASKSP-R (McClain et al., 2020)					
Guessing Rate			0.57	0.25	0–1
Autism Knowledge Percent Correct (Don’t Know/Missing Responses Incorrect)			0.54	0.13	0.20–0.92
Autism Knowledge Percent Correct (Don’t Know/Missing Responses Randomized)			0.60	0.11	0.32–0.92
ASK-Q (Harrison et al., 2017a)					
Guessing Rate			0.66	0.30	0–1
Autism Knowledge Percent Correct (Don’t Know/Missing Responses Incorrect)			0.87	0.06	0.6-1
Autism Knowledge Percent Correct (Don’t Know/Missing Responses Randomized)			0.89	0.04	0.69-1

*Note* ^This variable is measured on an ordinal scale, not a ratio scale. See full description under Measures

### Measures

#### Autism Spectrum Knowledge Scale Professional Version–Revised (ASKSP-R)

The ASKSP-R (McClain et al., 2020) is a 25-item multiple choice assessment created to measure autism knowledge within professional populations who are likely to work with autistic individuals. There are four multiple choice response options for each question, in addition to a “don’t know” response option. It was revised from the Autism Spectrum Knowledge Scale Professional Version (ASKSP; McClain et al., 2019), a unidimensional 33-question true/false measure. The ASKSP-R was created to remedy the substandard psychometrics and ceiling effect of the original instrument. The unidimensional ASKSP-R was validated in a prior publication (McClain et al., 2020) with this sample of school-based professionals (*n* = 427), and it showed acceptable internal consistency (KR20 = 0.070 raw, 0.71 standardized; λ6 = 0.73). The ASKSP-R has since been validated with interprofessional trainees (α = 0.76; Bono et al., 2022).

#### Autism Stigma and Knowledge Questionnaire (ASK-Q)

The ASK-Q (Harrison et al., 2017a) is a 49-item agree/disagree/don’t know measure created to assess both autism knowledge and stigma in the general population. As the first question on this assessment gathers background information on the participant, the remaining 48 questions objectively assess autism knowledge. The ASK-Q has been validated with undergraduate students (*n* = 313) and the general population (*n* = 304) and is a reliable measure (α = 0.88). The ASK-Q has a four-factor structure that measures autism knowledge in the areas of diagnosis/symptoms, etiology, treatment, and stigma.

#### Demographics and Autism Experience Questionnaire

Participants completed a brief questionnaire on demographic information (e.g., discipline, gender, degree, number of years practicing) and autism experience. Participants rated their self-perceived knowledge of autism and confidence in providing school-based services to students with autism on a scale from 1 (no knowledge/not confident) to 10 (extensive knowledge/extremely confident). They indicated the degree to which their various educational experiences and practical/clinical experiences were related to autism on a scale from 1 (none) to 4 (entire focus). Also, participants noted whether they knew someone with autism outside of their professional/training experiences, and they used an ordinal scale to approximate the number of autistic students they had worked with, from 1 (0 students) to 7 (> 50 students).

### Procedure

Data were collected as part of a larger project focused on autism knowledge. Participants completed all study tasks electronically via Qualtrics. Participants completed a brief demographic questionnaire and two psychometrically well-established autism knowledge questionnaires following consent. Participants were not given a time limit in which they needed to complete the study.

### Data Analyses

Four hierarchical linear regressions were conducted for both the ASKSP-R (McClain et al., 2020) and the ASK-Q (Harrison et al., 2017a) autism knowledge questionnaires.

*Guessing Rate Regression:* The dependent variable for this regression was guessing rate. Guessing rate was calculated using the following formula, slightly modified from Mondak and Canache (2004):

$$\frac{\text{Number\,of\,incorrect\,responses}} {\text{Number\,of\,incorrect\,responses} + \,\text{Number\,of\,``don't\,know"\,responses\,} + \text{Number\,of\,missing\,responses}}$$

Predictor variables were entered into the regression in three blocks. The first block of predictors focused on demographic and control variables. Discipline (0 = audiologist or speech-language pathologist, 1 = school psychologist), gender (0 = female, 1 = male), degree (0 = master’s or specialist’s, 1 = doctorate), and number of years practicing in any setting (centered) were entered in the first block of predictors. The second block of predictors focused on perceived autism expertise. Self-perceived knowledge of autism (centered) and confidence in providing school-based services to students with autism (centered) were entered in the second block of predictors. The third block of predictors focused on actual autism expertise. Educational experiences related to autism (averaged across experiences and centered), practical/clinical experiences related to autism (averaged across experiences and centered), knowing someone with autism outside of professional/training experiences (0 = knowing someone with autism personally, 1 = not knowing anyone with autism personally), and the number of students with autism that the participant has worked with (centered) were entered in the third block of predictors. Centered variables were centered at the mean.

*All Autism Knowledge Regressions*: For all autism knowledge regressions, the dependent variable was the percentage of correct responses on the autism knowledge questionnaire. The predictor variables/blocks were the same as described in the regression for guessing rate, with the exceptions noted below. The three autism knowledge regressions differed in their approach to correcting for (or not correcting for) guessing rate, as described below.

*Autism Knowledge Regression #1*: Autism knowledge questions that participants did not know or skipped were marked as incorrect, and there was no correction for guessing rate.

*Autism Knowledge Regression #2*: Autism knowledge questions that participants did not know or skipped were again marked as incorrect. However, there was a correction for guessing rate, with guessing rate included as a predictor variable in the first block of predictors.

*Autism Knowledge Regression #3*: Autism knowledge questions that participants did not know or skipped were randomly assigned a response. As such, the percentage of correct responses on the autism knowledge questionnaire included both (1) responses that participants originally answered correctly and (2) responses that participants originally did not know or skipped, but wherein the randomly assigned response matched the correct answer.

## Results

For the ASKSP-R (McClain et al., 2020), participants received an average score of 54% (*SD* = 13%) when questions that participants did not know or skipped were considered to be incorrect; the average score increased to 60% (*SD* = 11%) when these questions were randomly assigned a response. The average guessing rate for the ASKSP-R was 57% (*SD* = 25%), indicating that participants took a guess on 57% of the questions marked as incorrect. For the ASK-Q (Harrison et al., 2017a), participants received an average score of 87% (*SD* = 6%) when questions that participants did not know or skipped were considered to be incorrect; the average score increased to 89% (*SD* = 4%) when these questions were randomly assigned a response. The average guessing rate for the ASK-Q was 66% (*SD* = 30%), indicating that participants took a guess on 66% of the questions marked as incorrect. See Table 1 for more detailed descriptive statistics. Regression results are reported for the ASKSP-R in Table 2 and for the ASK-Q in Table 3.

**Table 2:** Hierarchical linear regressions for ASKSP – R (McClain et al., 2020)

Variable	Guessing Rate		Autism Knowledge #1 (No Correction for Guessing Rate)		Autism Knowledge #2 (Guessing Rate as Predictor Variable)		Autism Knowledge #3 (Don’t Know/Missing Responses Randomized)	
	*B*	β	*B*	β	*B*	β	*B*	β
Step 1								
Discipline	0.06 (0.03)*	0.12	0.06 (0.01)*	0.24	0.06 (0.01)*	0.21	0.04 (0.01)*	0.19
Gender	0.03 (0.05)	0.03	-0.02 (0.02)	-0.05	-0.03 (0.02)	-0.06	-0.03 (0.02)	-0.07
Degree	0.02 (0.04)	0.03	< 0.01 (0.02)	0.01	< 0.01 (0.02)	<-0.01	< 0.01 (0.02)	<-0.01
Years Practicing	< 0.01 (< 0.01)*	0.12	<-0.01 (< 0.01)	-0.07	<-0.01 (< 0.01)	-0.10	<-0.01 (< 0.01)	-0.10
Guessing Rate^	---	---	---	---	0.13 (0.02)*	0.25	---	---
Step 2								
Perceived Autism Knowledge	0.03 (0.02)	0.15	0.01 (0.01)	0.06	< 0.01 (0.01)	0.03	< 0.01 (0.01)	0.02
Confidence Providing Services to Autistic Students	-0.01 (0.01)	-0.05	0.01 (0.01)	0.07	0.01 (0.01)	0.08	0.01 (0.01)	0.07
Step 3								
Educational Experiences Related to Autism	0.04 (0.04)	0.07	0.01 (0.02)	0.03	< 0.01 (0.02)	0.01	< 0.01 (0.02)	0.01
Practical/Clinical Experiences Related to Autism	0.07 (0.03)*	0.13	0.01 (0.02)	0.06	0.01 (0.02)	0.02	< 0.01 (0.01)	0.02
Not Personally Knowing Someone with Autism	-0.07 (0.03)*	-0.10	-0.03 (0.02)	-0.08	-0.02 (0.02)	-0.05	-0.01 (0.01)	-0.02
Number of Autistic Students Worked With	0.01 (0.01)	0.05	0.02 (< 0.01)*	0.20	0.01 (< 0.01)*	0.18	0.01 (< 0.01)*	0.21

*Note* Standard errors are in parentheses. *p < 0.05. ^This variable was only included as a predictor in the Autism Knowledge #2 (Guessing Rate as Predictor Variable) regression

**Table 3:** Hierarchical linear regressions for ASK – Q (Harrison et al., 2017a)

Variable	Guessing Rate		Autism Knowledge #1 (No Correction for Guessing Rate)		Autism Knowledge #2 (Guessing Rate as Predictor Variable)		Autism Knowledge #3 (Don’t Know/Missing Responses Randomized)	
	*B*	β	*B*	β	*B*	β	*B*	β
Step 1								
Discipline	0.05 (0.03)	0.09	<-0.01 (0.01)	-0.04	-0.01 (0.01)	-0.07	-0.01 (0.01)	-0.06
Gender	-0.01 (0.06)	-0.01	-0.01 (0.01)	-0.03	-0.01 (0.01)	-0.03	-0.01 (0.01)	-0.06
Degree	-0.01 (0.04)	-0.01	0.02 (0.01)	0.10	0.02 (0.01)*	0.09	0.02 (0.01)*	0.16
Years Practicing	<-0.01 (< 0.01)	<-0.01	< 0.01 (< 0.01)	-0.02	<-0.01 (< 0.01)	-0.01	<-0.01 (< 0.01)	-0.02
Guessing Rate^	---	---	---	---	0.10 (0.01)*	0.51	---	---
Step 2								
Perceived Autism Knowledge	0.05 (0.02)*	0.23	0.01 (< 0.01)	0.16	< 0.01 (< 0.01)	0.06	< 0.01 (< 0.01)	0.02
Confidence Providing Services to Autistic Students	< 0.01 (0.02)	0.01	< 0.01 (< 0.01)	0.04	< 0.01 (< 0.01)	0.05	< 0.01 (< 0.01)	0.08
Step 3								
Educational Experiences Related to Autism			< 0.01 (0.01)	0.03	< 0.01 (0.01)	0.02	<-0.01 (0.01)	-0.02
Practical/Clinical Experiences Related to Autism			< 0.01 (0.01)	<-0.01	< 0.01 (0.01)	<-0.01	< 0.01 (0.01)	<-0.01
Not Personally Knowing Someone with Autism			-0.02 (0.01)*	-0.12	-0.02 (0.01)*	-0.12	-0.02 (0.01)*	-0.14
Number of Autistic Students Worked With			0.01 (< 0.01)*	0.16	< 0.01 (< 0.01)*	0.10	< 0.01 (< 0.01)*	0.15

*Note* Standard errors are in parentheses. *p < 0.05. ^This variable was only included as a predictor in the Autism Knowledge #2 (Guessing Rate as Predictor Variable) regression

### ASKSP-R (McClain et al., 2020)

*Guessing Rate Regression: *The first block of predictors (demographics) significantly predicted guessing rate, *F*(4, 389) = 4.24, *p* < 0.01. The second block of predictors (perceived autism expertise), Δ*F*(2, 387) = 9.56, *p* < 0.01, and the third block of predictors (actual autism expertise), Δ*F*(4, 383) = 3.96, *p* < 0.01, significantly predicted guessing rate above and beyond the prior block of predictors, such that the third block of predictors was retained as the final regression model. Participants whose discipline was school psychology, *t*(383) = 2.18, *p* = 0.03, who had more years practicing in any setting, *t*(383) = 2.17, *p* = 0.03, who had more practical/clinical experiences related to autism, *t*(383) = 2.15, *p* = 0.03, and who personally knew someone with autism, *t*(383) = -2.07, *p* = 0.04, had a higher guessing rate.

*Autism Knowledge Regression #1 (no guessing rate correction):* The first block of predictors significantly predicted autism knowledge, *F*(4, 389) = 8.98, *p* < 0.01. The second block of predictors, Δ*F*(2, 387) = 11.97, *p* < 0.01, and the third block of predictors, Δ*F*(4, 383) = 5.22, *p* < 0.01, significantly predicted autism knowledge above and beyond the prior block of predictors, such that the third block of predictors was retained as the final regression model. Participants whose discipline was school psychology, *t*(383) = 4.78, *p* < 0.01, and who had worked with more students with autism, *t*(383) = 3.76, *p* < 0.01, had greater autism knowledge.

*Autism Knowledge Regression #2 (guessing rate as a predictor variable):* The first block of predictors significantly predicted autism knowledge, *F*(5, 388) = 16.66, *p* < 0.01. The second block of predictors, Δ*F*(2, 386) = 7.28, *p* < 0.01, and the third block of predictors, Δ*F*(4, 382) = 4.04, *p* < 0.01, significantly predicted autism knowledge above and beyond the prior block of predictors, such that the third block of predictors was retained as the final regression model. Participants who had a higher guessing rate, *t*(382) = 5.32, *p* < 0.01, whose discipline was school psychology, *t*(382) = 4.33, *p* < 0.01, and who had worked with more students with autism, *t*(382) = 3.66, *p* < 0.01, had greater autism knowledge.

*Autism Knowledge Regression #3 (don’t know/missing responses randomized):* The first block of predictors significantly predicted autism knowledge, *F*(4, 389) = 5.99, *p* < 0.01. The second block of predictors, Δ*F*(2, 387) = 5.40, *p* = 0.01, and the third block of predictors, Δ*F*(4, 383) = 3.87, *p* < 0.01, significantly predicted autism knowledge above and beyond the prior block of predictors, such that the third block of predictors was retained as the final regression model. Participants whose discipline was school psychology, *t*(383) = 3.67, *p* < 0.01, and who had worked with more students with autism, *t*(383) = 3.79, *p* < 0.01, had greater autism knowledge.

### ASK-Q (Harrison et al., 2017a)

*Guessing Rate Regression*^1^ The first block of predictors marginally predicted guessing rate, *F*(4, 383) = 2.06, *p* = 0.09. The second block of predictors, Δ*F*(2, 381) = 10.43, *p* < 0.01, significantly predicted guessing rate above and beyond the first block of predictors. The third block of predictors, Δ*F*(4, 377) = 0.98, *p* = 0.42, did not significantly predict guessing rate above and beyond the second block of predictors, such that the second block of predictors was retained as the final regression model. Participants with a greater self-perceived knowledge of autism, *t*(381) = 2.68, *p* = 0.01, had a higher guessing rate.

*Autism Knowledge Regression #1 (no guessing rate correction):* The first block of predictors did not significantly predict autism knowledge, *F*(4, 384) = 1.46, *p* = 0.21. The second block of predictors, Δ*F*(2, 382) = 15.10, *p* < 0.01, and the third block of predictors, Δ*F*(4, 378) = 4.23, *p* < 0.01, significantly predicted autism knowledge above and beyond the prior block of predictors, such that the third block of predictors was retained as the final regression model. Participants who personally knew someone with autism, *t*(378) = -2.45, *p* = 0.02, and who had worked with more students with autism, *t*(378) = 2.95, *p* < 0.01, had greater autism knowledge.

*Autism Knowledge Regression* #2^1^*(guessing rate as a predictor variable):* The first block of predictors significantly predicted autism knowledge, *F*(5, 382) = 34.52, *p* < 0.01. The second block of predictors, Δ*F*(2, 380) = 6.56, *p* < 0.01, and the third block of predictors, Δ*F*(4, 376) = 3.47, *p* = 0.01, significantly predicted autism knowledge above and beyond the prior block of predictors, such that the third block of predictors was retained as the final regression model. Participants who had a higher guessing rate, *t*(376) = 11.71, *p* < 0.01, had a doctorate degree, *t*(376) = 2.04, *p* = 0.04, personally knew someone with autism, *t*(376) = -2.70, *p* = 0.01, and who had worked with more students with autism, *t*(376) = 2.22, *p* = 0.03, had greater autism knowledge.

*Autism Knowledge Regression #3 (don’t know/missing responses randomized): *The first block of predictors significantly predicted autism knowledge, *F*(4, 384) = 3.00, *p* = 0.02. The second block of predictors, Δ*F*(2, 382) = 4.88, *p* = 0.01, and the third block of predictors, Δ*F*(4, 378) = 4.22, *p* < 0.01, significantly predicted autism knowledge above and beyond the prior block of predictors, such that the third block of predictors was retained as the final regression model. Participants who had a doctorate degree, *t*(378) = 3.12, *p* < 0.01, personally knew someone with autism, *t*(378) = -2.72, *p* = 0.01, and who had worked with more students with autism, *t*(378) = 2.77, *p* = 0.01, had greater autism knowledge.

## Discussion

The current study is the first to examine the impact of the “don’t know” response option on autism knowledge assessments. Results show that participants’ tendency to guess, as opposed to selecting the “don’t know” response option, significantly impacts the measurement of autism knowledge. Predictors of guessing rate and autism knowledge are discussed in greater detail below, and recommendations are given for designing future autism knowledge assessments and using pre-existing autism knowledge assessments with a “don’t know” response option.

### Guessing Rate

For the ASKSP-R (McClain et al., 2020), school psychologists, participants who had more years practicing in any setting, participants who had more practical/clinical experiences related to autism, and participants who personally knew someone with autism had a higher guessing rate. While the last two of these variables directly index autism experience, the first two of these variables may also indirectly index autism experience. School psychologists may receive more extensive autism training than audiologists and speech-language pathologists, and participants who have been practicing longer may have had more experience, even if indirect or limited experience, with autism or autistic individuals. These results suggest that participants with more autism experience, or more professional experience in general, may be more likely to guess on an autism knowledge assessment. For the ASK-Q (Harrison et al., 2017a), participants with greater self-perceived knowledge of autism had a higher guessing rate. As perceived autism knowledge may not be related to actual autism knowledge (McMahon et al., 2020), this result suggests that individuals who perceive themselves to be more knowledgeable about autism may actually receive higher scores on autism knowledge assessments, simply due to their greater propensity to guess.

Of note, different variables predicted guessing rate for the ASKSP-R (McClain et al., 2020) and the ASK-Q (Harrison et al., 2017a). The ASKSP-R has four response options (in addition to the “don’t know” option), with an average guessing rate of 57% (see Table 1). The ASK-Q has two response options (in addition to the “don’t know” option), with an average guessing rate of 66%. Participants may be more likely to guess on an easier questionnaire (with a 50% likelihood of guessing correctly) compared to a more challenging questionnaire (with a 25% likelihood of guessing correctly). Thus, predictors of guessing rate may depend on the difficulty of the questionnaire and/or the likelihood of guessing correctly on the questionnaire.

### Autism Knowledge

Guessing rate was the strongest predictor of autism knowledge across both the ASKSP-R (McClain et al., 2020) and the ASK-Q (Harrison et al., 2017a) assessments (see the standardized coefficient β column for Autism Knowledge Regression #2 on Tables 2 and 3). Participants who were more likely to guess on autism knowledge assessments received higher autism knowledge scores. Participants who were less likely to guess on autism knowledge assessments received lower autism knowledge scores. As such, two participants with the same underlying knowledge of autism, but with different guessing rates, would receive different autism knowledge scores. The “don’t know” response option, therefore, reduces the validity of these assessments, as guessing rate is a systematic (nonrandom) source of variance in final autism knowledge scores (Mondak, 1999).

For both the ASKSP-R and ASK-Q assessments, participants who worked with more students with autism scored higher in autism knowledge, even after controlling for other variables related to autism experience. Whereas many of the autism experience variables in this study indexed *depth* of experience (e.g., educational experiences, practical/clinical experiences), this variable may have indexed *breadth* of experience. Professionals who have spent considerable time working with a few select autistic individuals or a specific subgroup within the autism spectrum (e.g., autistic individuals with an intellectual disability) may not have the *breadth* of experience required to answer some general questions about the autism spectrum. Professionals who have worked with more students with autism may be more familiar with the heterogeneity across the autism spectrum, and therefore better equipped to answer general autism knowledge questions.

For the ASKSP-R (McClain et al., 2020), school psychologists showed greater autism knowledge than audiologists and speech-language pathologists. This result may indicate that school psychologists receive more autism-specific training than audiologists and speech-language pathologists. Conversely, as this assessment was designed by school psychologists, the questions on this assessment may skew toward autism knowledge more relevant for school psychologists compared to audiologists and speech-language pathologists.

For the ASK-Q (Harrison et al., 2017a), participants who personally knew someone with autism had greater autism knowledge. While the majority of the autism experience variables in this study indexed professional experience, this result demonstrates that personal autism experience is equally important to consider and should be a focus of future research. In addition, in the second regression (guessing rate as a predictor variable) and third regression (don’t know/missing responses randomized), participants who had a doctorate degree, compared to a master’s or specialist’s degree, also showed greater autism knowledge. As such, greater education positively impacts autism knowledge. Additionally, it is worth noting that this result only occurred in the second and third regressions, which corrected for guessing rate, and was not observed in the first regression. In order to accurately assess the relationship between predictor variables and autism knowledge, it may be critical to correct for guessing rate.

### Recommendations for Autism Knowledge Assessments

Previous studies in the broader social science literature (e.g., Miller & Orr, 2008; Mondak, 1999, 2001; Mondak & Anderson, 2004; Mondak & Davis, 2001) suggest caution in using the “don’t know” response option on knowledge assessments. While the “don’t know” response option is currently included in several autism knowledge assessments (e.g., Harrison et al., 2017a, b; McClain et al., 2020), the results of the present study, in combination with the broader literature, suggest that this response option should be eliminated from autism knowledge assessments.

We provide the following recommendations for researchers who wish to revise previously developed autism knowledge assessments, design new autism knowledge assessments, or use pre-existing autism knowledge assessments that include a “don’t know” response option:

1. When designing new autism knowledge assessments, researchers should not include a “don’t know” response option. Instead, researchers should encourage participants to try their best when responding to each autism knowledge question, even if uncertain of the answer.
2. If researchers wish to use a pre-existing autism knowledge assessment that includes a “don’t know” response option, we recommend that the “don’t know” option be removed from this assessment, if possible. There are two caveats to note about this recommendation. First, removing the “don’t know” option will change the reported psychometric properties of the assessment. Although this change is expected to improve the validity of the assessment, the psychometric properties of the assessment will need to be reevaluated, without the “don’t know” response option. Second, if researchers wish to compare their data with pre-existing data using the original version of the assessment, researchers should then consider using the original version of the assessment to allow for this comparison.
3. If researchers have a theoretical rationale for using the original assessment with the “don’t know” response option, they should consider analyzing the data without a correction for guessing rate (e.g., allowing for comparison with other data sets) *and* with a correction for guessing rate. Researchers can correct for guessing rate by (1) calculating guessing rate using the formula presented in the Methods section of this paper and including guessing rate in the analysis as a control variable or (2) randomly assigning a guess to any “don’t know” or skipped responses.
4. If researchers are interested in participants’ metacognitive awareness, or the degree to which they can accurately assess their own autism knowledge, they should ask participants to provide a response for all questions *and* rate their level of confidence in each response. In this way, autism knowledge and metacognitive awareness can be assessed separately (see McMahon et al., 2016; Sawyer et al., 2014; Wilkinson et al., 2010 for examples). Researchers will be able to assess whether participants feel as though they don’t know or are guessing on their response *and* collect data on autism knowledge that is unaffected by guessing rate.

Finally, it is worth explicitly noting that these recommendations encourage participants to take educated guesses on all knowledge questions. As such, participants will sometimes randomly guess the correct answer, without actually knowing that answer. All participants, therefore, will have knowledge scores that are slightly elevated due to random guesses. While this is a limitation of this approach, it is superior to the alternative approach, as random error, rather than systematic error, is included in the measurement of autism knowledge (e.g., Mondak, 1999). In addition, this approach allows participants with partial knowledge about a question to use that partial knowledge in their response, thus more effectively differentiating between participants with partial versus no knowledge. By following the recommendations provided in this section, researchers will be able to more accurately assess autism knowledge, as well as separately assess metacognitive awareness of autism knowledge, if this is a genuine construct of interest.

### Limitations and Future Directions

There are some limitations that must be considered when interpreting the implications of this study. First, participants were recruited through audiology, school psychology, and speech-language pathology professional organizations. This may have limited the findings in various ways. First, typically only members of such professional organizations are included in advertisements about study participation, thus limiting the potential pool of participants. We relied on professional associations to disseminate information about the study, and thus the authors did not have the ability to try to obtain specific participants (e.g., from certain regions or that represent various identities). In addition, this study looked at three different disciplines (audiology, school psychology, and speech-language pathology), which are not representative of the diverse disciplines that interact and work with autistic populations. Furthermore, we recruited participants who work in educational settings. Although it is important to understand autism knowledge in educational settings, individuals in other contexts may have different levels of knowledge based on diverse roles and experiences. It is crucial to examine autism knowledge in diverse populations, such as law enforcement and medical personnel. Additionally, school-based professionals vary considerably in their educational experiences and training related to autism, and the relationship between guessing rate and autism knowledge may differ in participant samples with more homogenous levels of autism knowledge (e.g., most participants are autism novices or experts).

This study also used two different autism knowledge measures. These measures were selected based on their strong psychometric properties and use of the “don’t know” response category. However, other autism knowledge measures with “don’t know” response categories may perform differently. In particular, the different pattern of responses across the ASKSP-R and ASK-Q assessments suggests that the difficulty of the questionnaire may affect participants’ propensity to guess or utilize the “don’t know” response option.

In this study, personally knowing someone with autism was roughly dichotomized as a yes/no variable. While participants did indicate their relationship with this person (e.g., immediate family member, coworker), it was challenging to use this information to measure degree of personal contact. Relationship status does not necessarily index relationship closeness. For instance, a person might collaborate with a coworker daily or might rarely see a coworker. Also, the number of autistic people that someone knows personally may not directly correspond to personal autism expertise. An individual who knows one person with autism well might have more personal expertise with autism than someone who marginally knows three autistic people. Relatedly, in this study, we did not ask participants to indicate whether they themselves were autistic. Future research should more precisely operationalize personal autism experience, including the firsthand experience of having an autism diagnosis.

Finally, this study used an ordinal scale to approximate the number of autistic students that participants had worked with as a school-based professional. Participants may have had difficulty accurately estimating this number, particularly participants who have been working in the field for many years. As this variable emerged as a significant predictor of autism knowledge on both the ASKSP-R and ASK-Q assessments, this construct should be examined more carefully and operationalized more precisely in future research. If possible, it would be advantageous to review medical or school records that could provide an objective index of the number of autistic students that professionals have worked with over their career. Additionally, it would be helpful to have an estimate of the amount of time professionals spend with each student, as some school-based professionals are more likely to have an extended relationship with autistic students (e.g., providing ongoing services) and other school-based professionals may be more likely to have singular interactions with autistic students (e.g., conducting an initial assessment). As the number of autistic students worked with was the only variable (aside from guessing rate) to significantly predict autism knowledge across both assessments, it may be critical to index breadth of experience working with different autistic individuals. Future research should consider alternative methods for measuring breadth of autism experience or familiarity with the heterogeneity of the autism spectrum.

This study investigated two specific autism knowledge measures (ASKSP-R and ASK-Q). Future research on additional autism knowledge measures is warranted, and revision of existing measures to exclude the “don’t know” response is indicated. Future research should also continue to investigate potential predictors of autism knowledge, as other predictors not directly measured in this study (e.g., experience working with autistic individuals with varying support needs) may be important to consider. Psychometrically strong autism knowledge measures should be more regularly integrated into research investigating autism identification and intervention services, family partnerships, advocacy, and policy. A stronger understanding of the potential benefits of autism knowledge and service delivery can inform future professional learning, training, and certification processes. Greater autism knowledge will ultimately contribute to improved outcomes for children and families with autism, and likely higher efficacy and confidence of professionals that are serving this population.

This study indicates that the use of the “don’t know” response may not be beneficial on autism knowledge assessments. However, respondents may believe they do not know the response to the item, and their beliefs regarding their knowledge are important to measure. Qualitative methods to obtain information pertaining to respondents’ autism knowledge may help determine aspects contributing to participants’ understanding. Additionally, quantitative measures that ask participants to index their degree of certainty in their response to each question can provide valuable metacognitive feedback, without introducing error into the measurement of autism knowledge. Future autism knowledge measurement would also be strengthed by increasing multimodal assessment techniques, validating measures among various professionals, and examining such measures among diverse populations and demographic groups.

## Footnotes

1 Note that one participant received a score of 100% on the ASK-Q. As this participant didn’t have any Incorrect, ”don’t know”, or missing responses, a guessing rate could not be calculated for this participant. This participant is excluded from the Guessing Rate Regression and the Autism Knowledge Regression #2 for the ASK-Q.
